# Novel *PRPF31* mutations associated with Chinese autosomal dominant retinitis pigmentosa patients

**Published:** 2012-12-14

**Authors:** Fei Xu, Ruifang Sui, Xiaofang Liang, Hui Li, Ruxin Jiang, Fangtian Dong

**Affiliations:** Department of Ophthalmology, Peking Union Medical College Hospital, Peking Union Medical College and Chinese Academy of Medical Sciences

## Abstract

**Purpose:**

To identify the mutations in the pre-mRNA processing factor 31 homolog (*PRPF31*) gene in Chinese families with autosomal dominant retinitis pigmentosa (adRP) and to characterize the clinical features of those patients who were found to have mutations in the *PRPF31* gene.

**Methods:**

Detailed ocular examinations were performed, and genomic DNA was isolated by standard methods for genetic diagnosis. Probands from each family were screened for mutations in the *PRPF31* gene that was known to cause adRP. Cosegregation analysis was performed in the available family members. Linkage analysis was performed for one missense mutation to calculate the likelihood of its pathogenicity. Two hundred unrelated, healthy Chinese subjects were screened to exclude nonpathogenic polymorphisms.

**Results:**

Forty Chinese families with adRP were selected by an analysis of pedigrees. We identified four mutations (c.196_197delAA, c.544_618del75bp, c.615delC, and c.895T>C) in total, and three deletions were novel. Cosegregation analysis of the available family members (20 patients and 17 unaffected family members) revealed that each index patient and all affected family members showed a heterozygous mutation in the *PRPF31* gene. In two families, incomplete penetrance was observed. Linkage analysis achieved the maximum LOD score of c.895T>C is 2.09, achieved at θ=0. The four probands with *PRPF31* mutations showed classical signs of RP, with relatively preserved central vision and severe visual field constriction.

**Conclusions:**

Our studies extended the mutation spectrum of *PRPF31,* and mutations in *PRPF31* were found at a relatively high frequency (10%, 4 of 40 adRP families) in our cohort.

## Introduction

Retinitis pigmentosa (RP), broadly defined to include nonsyndromic and syndromic forms, is a group of clinically and genetically heterogeneous, inherited retinal diseases characterized by the progressive degeneration of the photoreceptors, which eventually leads to blindness. Nonsyndromic RP is the most common inherited form of severe retinal degeneration, with a prevalence of approximately 1/4,000 RP cases worldwide [1]. Nonsyndromic cases can be inherited as an autosomal-dominant (20%–25%), autosomal-recessive (15%–20%), X-linked recessive (10%–15%), or sporadic/simplex (30%) trait [2]. The typical clinical manifestations of nonsyndromic RP include the presence of night blindness, peripheral visual field defects, lesions in the fundus (bone spicule-like retinal pigmentary deposits and chorioretinal atrophy), and a severely reduced or nondetectable electroretinogram (ERG) [1].

To date, mutations in 23 different genes have been associated with autosomal dominant retinitis pigmentosa (adRP; RetNet) genes. One of these is the gene pre-mRNA processing factor 31 homolog (*PRPF31*), which is localized on chromosome 19q13.4 (RP11). Interestingly, *PRPF31* is one of six pre-mRNA splicing factors identified as causing adRP. The *PRPF31* gene comprises 14 exons spanning approximately 18 kb of genomic DNA and encodes a protein of 499 amino acids. The *PRPF31* gene is ubiquitously expressed, including in the neural tissues, brain, and retina [3]. This 61-kDa protein is required for the formation of the U4/U6·U5 tri-small nuclear ribonucleoprotein spliceosome complex, which serves to ensure the accurate and efficient splicing of pre-mRNA [4].

According to previous reports, the prevalence of *PRPF31* mutations ranges from 2% to 10% in adRP cohorts from various geographical origins [5-10]. To date, more than 50 adRP-associated *PRPF31* mutations have been identified, including missense/nonsense mutations, splicing mutations, deletions/insertions, small indels, and complex mutations (HGMD). Most of the previous genetic studies on *PRPF31* were performed in the western world, and only limited data from Chinese patients are available [9,11-13]. The purpose of this study is to assess the genotypes and phenotypes of Chinese adRP patients with *PRPF31* mutations.

## Methods

### Recruitment of subjects

All participants were identified at Peking Union Medical College Hospital. The diagnosis of RP was based on the presence of night blindness, typical fundus findings (characteristic retinal pigmentation, vessel attenuation, and various degrees of retinal atrophy), the severe loss of peripheral visual field, and abnormal electroretinogram findings (dramatic diminution in amplitudes or the complete absence of response). To be included in our adRP cohort, the patients had to have at least three generations of affected individuals or two generations with evidence of male-to-male transmission. Written informed consent was obtained from all participating individuals. This study was approved by the Institutional Review Board of Peking Union Medical College Hospital and adhered to the tenets of the Declaration of Helsinki and the Guidance on Sample Collection of Human Genetic Diseases by the Ministry of Public Health of China.

### Clinical evaluations

For each patient, a full medical and family history was taken and an ophthalmological examination was performed. Each underwent a standard ophthalmic examination: best correct visual acuity according to Snellen charts, slit-lamp biomicroscopy, dilated indirect ophthalmoscopy, fundus photography if possible, and visual field tests (Octopus; Interzeag, Schlieren, Switzerland). Retinal structure was examined by using optical coherence tomography (OCT; Topcon, Tokyo, Japan). ERGs were performed (RetiPort ERG system; Roland Consult, Wiesbaden, Germany) using corneal “ERGjet” contact lens electrodes. The ERG protocol complied with the standards published by the International Society for Clinical Electrophysiology of Vision.

### Genetic studies

Genomic DNA was extracted from the peripheral white blood cells. We used 2ml blood which collected in EDTA vacutainer tubes. Genomic DNA was isolated from the peripheral leukocytes by using a QIAamp DNA Blood Midi Kit (Qiagen, Hilden, Germany) according to the manufacturer’s protocol. All 14 coding exons, including the intron–exon boundaries of the *PRPF31* gene, were amplified by PCR, using primers published previously [14]. After purification, amplicons were sequenced using both forward and reverse primers on an ABI 3730 Genetic Analyzer (ABI, Foster City, CA). Sequences were assembled and analyzed using Lasergene SeqMan software (DNASTAR, Madison, WI). The results were compared with the *PRPF31* reference sequence (GenBank accession number: NM_015629.3). The presence of novel variants was also investigated in 200 unrelated healthy Chinese control subjects by using direct sequencing to exclude nonpathogenic polymorphisms. Cosegregation analysis was performed in the available family members.

### Mutation identification

The pathogenicity of c.895T>C was measured by calculating the LOD score using the Linkage software package (version 5.2) with parameters during calculation were set as below. Allele frequencies of affection status were set at 0.9997 and 0.0003, according to the prevalence of RP; allele frequencies of the candidate mutation site (c.895T>C) were set at 0.995 and 0.005, according to Sullivan’s counting. The penetrance was set at 95%. MLINK program in Linkage software package was used in calculating the LOD score [15].

## Results

### Mutation analysis

**Figure 1:**
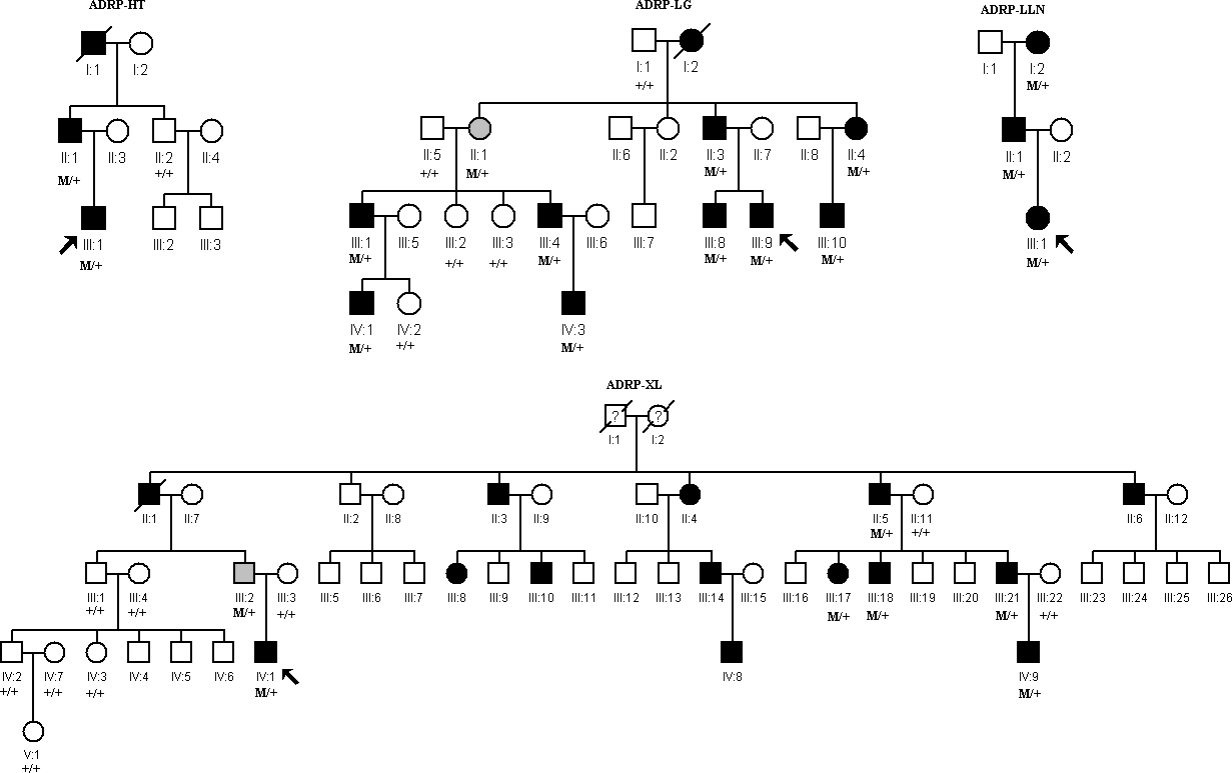
Pedigrees of four Chinese families with autosomal dominant retinitis pigmentosa associated with mutations in the *PRPF31* gene. Genotypes are shown beneath the symbols. Affected individuals are represented by black symbols, unaffected ones by unfilled, asymptomatic ones by gray; squares signify males, circles females. Arrows mark the index patients. M refers to the mutant allele, and + means normal allele.

**Figure 2:**
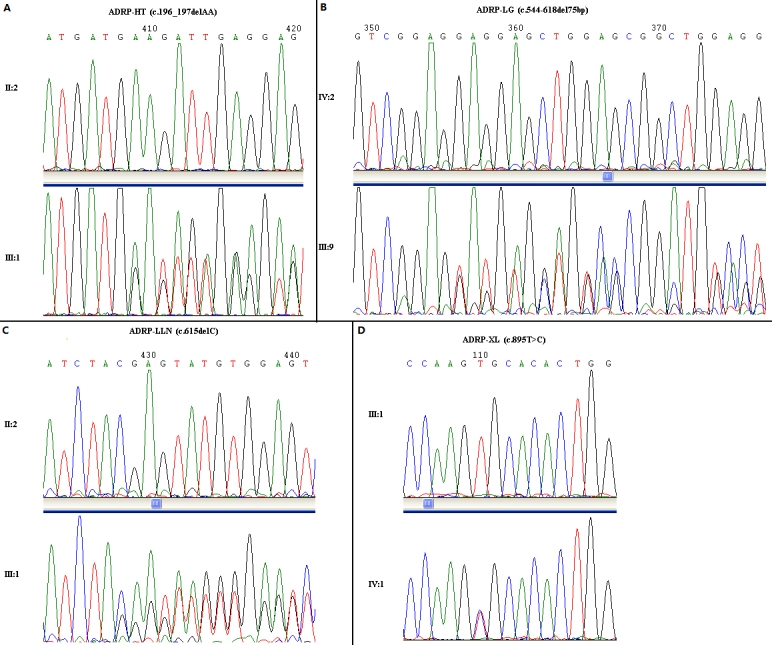
Sequencing results of the PRPF31 mutations in the four families. **A**: Family ADRP-HT; **B**: Family ADRP-LG; **C**: Family ADRP-LLN; **D**: Family ADRP-XL. The sequences of control subjects are shown in each top panel (**A**-**D**). The corresponding sequences of four probands are shown in the lower panel (**A**-**D**).

Upon complete sequence analysis of the coding and adjacent intronic regions of *PRPF31*, four mutations were detected in four out of 40 families, including three novel deletions and one previously reported missense mutation (Figure 1, Figure 2, Table 1). Two variants (c.895T>C and c.615delC) were not found in the 200 unrelated healthy Chinese controls. The three deletions were predicted to lead to premature stop codons. Cosegregation analysis revealed two families with incomplete penetrance (Table 1).

**Table 1:** Four *PRPF31* mutations identified in a Chinese adRP cohort.

Families	Exon	Nucleotide exchange	Protein effect	Information about penetrance
ADRP-HT	3	c.196_197delAA#	K66DfsX2	segregates
ADRP-LG	7	c.544_618del75 bp#	E182_E206del	incomplete
ADRP-LLN	7	c.615delC#	p.Y205*	segregates
ADRP-XL	9	c.895T>C [7]	p.C299R	incomplete

#: novel mutation.

### Mutation identification

Under the given parameters, the maximum LOD score of c.895T>C was 2.09, achieved at θ=0.

### Clinical assessment of four probands

**Figure 3:**
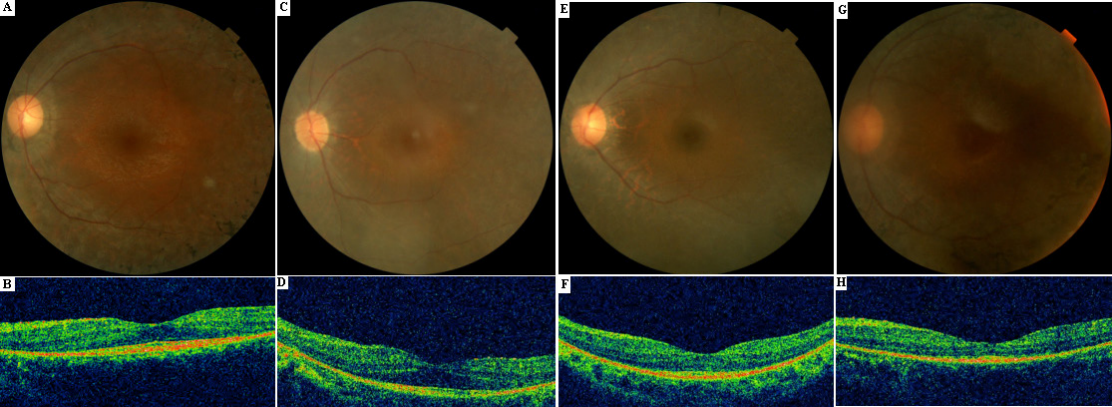
Fundus photographs(top panel) and optical coherence tomography images (lower panel) of four probands with mutations in the *PRPF31* gene: **A** and **B**: III:1 (ADRP-HT); **C** and **D**: III:9 (ADRP-LG); **E** and **F**: III:1 (ADRP-LLN); **G** and **H**: IV:1 (ADRP-XL). Typical retinitis pigmentosa appearance of the fundus can be seen (**A**, **C**, **E**, and **G**). Optical coherence tomography images (**B**, **D**, **F**, and **H**) reveal relatively preserved foveal lamination.

The clinical features of the four index patients with *PRPF31* mutations are summarized in Table 2 and Table 3. Three of them were males and one was female, with ages ranging from 26 to 29 years. Ages at the time of testing ranged from 24 to 27 years. The symptoms that led to the diagnosis were dominated by night blindness in all patients. Central visual acuity was relatively preserved. Anterior segment examinations showed a remarkable posterior subcapsular cataract in only one patient (IV:1, ADRP-XL). All patients showed severe visual-field constriction. Fundus examination with indirect ophthalmoscopy after full dilatation showed relatively normal disc color, varying amounts of pigment deposits, and different degrees of retinal pigment-layer atrophy (Figure 3). OCT examinations showed relatively preserved foveal lamination and the disappearance of the IS/OS layer, as well as the retinal pigment epithelium layer in the periphery (Figure 3). Full-field ERGs were nonrecordable under scotopic conditions (rods), and the cone responses were markedly hypovolted or nonrecordable in all these patients.

**Table 2:** Clinical data of probands from families with adRP due to *PRPF31* mutations.

Family/patient	Age at time of testing	Sex	Symptoms at time of diagnosis	BCVA OD/OS	Lens	Fundus examination
ADRP-HT/III:1	27	M	Night blindness	20/20 20/20	Clear	Normal disc color, multiple bone spicules in the mid- periphery retina
ADRP-LG/III:9	24	M	Night blindness	20/20 20/20	Clear	RPE atrophy in the temporal area of optic disc
ADRP-LLN/III:1	27	F	Night blindness	20/40 20/50	Clear	Multiple bone spicules in the mid- periphery retina
ADRP-XL/IV:1	27	M	Night blindness	20/25 20/25	Posterior subcapsular cataracts (OU)	Multiple bone spicules in the mid- periphery retina

The following abbreviation was used: BCVA: best corrected visual acuity.

**Table 3:** Functional data.

Family/patient	OCT	Visual field	Full field ERG (photopic and scotopic)
ADRP-HT/III:1	Preserved foveal lamination, disappearance of IS/OS layer in periphery	10° both horizontally and vertically	Non-recordable
ADRP-LG/III:9	Preserved foveal lamination, disappearance of IS/OS layer in periphery	10° both horizontally and vertically	Only residual cone responses
ADRP-LLN/III:1	Preserved foveal lamination, disappearance of IS/OS layer in periphery	Tunnel vision, <5° both horizontally and vertically	non-recordable in scotopic ERG and the cone responses were markedly reduced
ADRP-XL/IV:1	Preserved foveal lamination, disappearance of IS/OS layer in periphery	Tunnel vision, <5° both horizontally and vertically, more severe in the left eye	Non-recordable

### The phenotype of two obligate carriers

**Figure 4:**
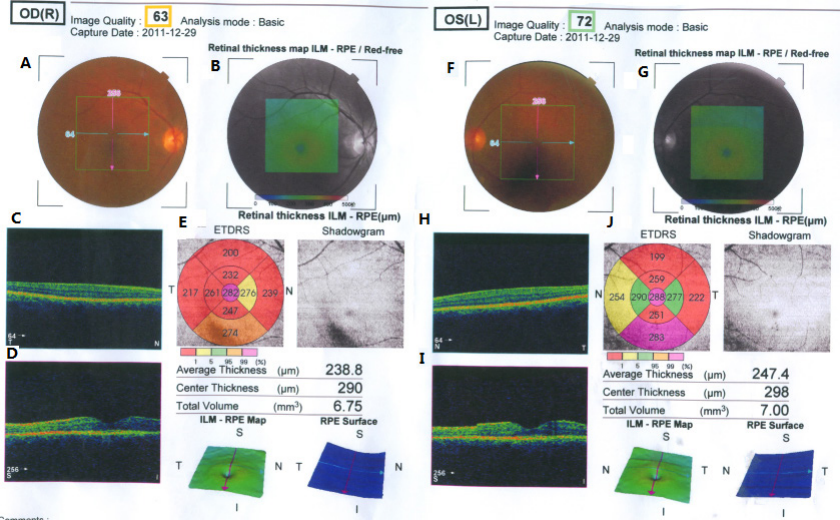
Fundus photographs (**A** and **F**) and optical coherence tomography images (**C**, **D**, **H**, and **I**) of both eyes for III:2 (ADRP-XL). Panel **B** and **G** indicated red-free fundus images. Panel **E** and **J** indicated the calculating results of macular thickness and total volume in the given areas. Right eye: panel **A**-**E**. Left eye: panel **F**-**J**. No signs of RP can be seen.

**Figure 5:**
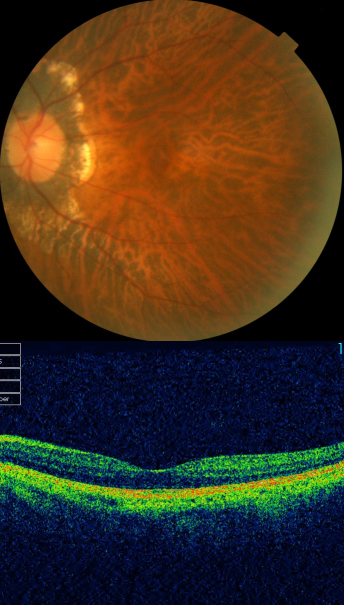
Fundus photograph and optical coherence tomography image of the right eye for II:1 (ADRP-LG). Fundus examination showed typical high myopic fundus changes including tilting of the optic disc, myopic conus, and tessellated fundus. Optical coherence tomography image revealed relatively normal macular lamination.

**Figure 6:**
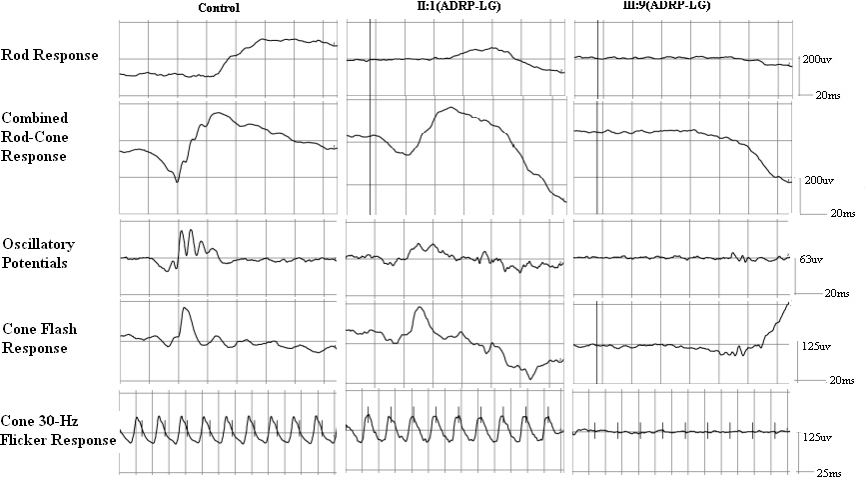
The comparison of full-field electroretinography between the proband (III:9) and the obligate carrier (II:1) in family ADRP-LG. Scotopic electroretinographs are nonrecordable, and other ERGs are severely reduced in III:9. Although the amplitudes of a-waves and b-waves of bright flash ERGs are slightly reduced, the other ERGs are within the normal range in the asymptomatic carrier II:1. The ERG protocol we used in this study complied with the standards published by the International Society for Clinical Electrophysiology of Vision (ISCEV). The light levels used to stimulate the eyes are available on the ISCEV Internet site.

In family ADRP-XL, an asymptomatic carrier (III:2) of the missense mutation (c.895T>C) in the *PRPF31* gene was identified, suggesting incomplete penetrance in this pedigree. The best corrected visual acuity of this asymptomatic carrier was 20/20 (in both eyes). The fundus and OCT exam did not reveal any changes typical of RP (Figure 4). Another family (ADRP-LG) with a relatively large deletion in the *PRPF31* gene (c.544_618del75bp) also exhibited incomplete penetrance. One of the proband’s paternal aunts (II:1, 57 years old, ADRP-LG) did not have any history of night blindness or visual field defects. Her best corrected visual acuity was 20/40 (right eye) and 20/25 (left eye). The refractions were −7.00 DS–2.25 DC × 153° (OD) and −5.00 DS − 1.25 DC × 50° (OS). Dilated fundus examination showed typical bilateral high myopic fundus changes (Figure 5). Detailed ophthalmic examination revealed relatively normal visual field and ERG tests (Figure 6), and therefore, a diagnosis of RP was ruled out for this person.

## Discussion

To determine the types and frequencies of disease-causing mutations in the *PRPF31* gene, we tested 40 Chinese families with adRP for mutations. We identified four different disease-causing mutations in four of 40 unrelated adRP families, including three novel deletions and one known missense mutation. To date, only few *PRPF31* variations have been reported to be missense mutations [10]. This also holds true for our study. As far as we know, this study is the largest series to date performed in Chinese adRP patients, and our findings expand the spectrum of *PRPF31* mutations. However, the mutation-screening protocols adopted in this study (PCR-based methods) cannot detect gross genomic rearrangements such as the large deletions that were found—using multiplex ligation-dependent probe amplification—to account for 2.5% of adRP families [16]. Thus, the 10% (four in 40 families) frequency of *PRPF31* mutations in our cohort is likely to be an underestimate. According to a previous study by Lim et al. [9], the variant frequency for the *PRPF31* gene (detected by direct sequencing) in a small cohort of Chinese adRP patients was estimated to be 11.1% (1/9 adRP families), indicating that *PRPF31*-associated adRP is relatively common in Chinese populations.

It is a common phenomenon that adRP patients with *PRPF31* mutations show “all or none” of the forms of incomplete penetrance, making it a high-priority candidate gene in such cases [6,7,10-12]. Consistent with previous reports, incomplete penetrance was observed in two of our four pedigrees, suggesting that most of the *PRPF31* mutations are indeed associated with incomplete penetrance. The mechanism presumed to explain this phenomenon is an allelic imbalance with the overexpression of the wild-type allele, compensating for the nonfunctional mutant allele in asymptomatic carriers [17,18]; however, the exact mechanism remains to be determined. Although one of the obligate carriers with a relatively large deletion of the *PRPF31* gene exhibited clear evidence of high myopia, given the high prevalence of myopia in East Asia, this finding could simply be a coincidence [19].

The missense mutation c.895T>C (p.C299R) was previously reported by Sullivan et al. in 2006: they predicted that it was probably a pathogenic mutation [7]. We identified this known mutation in a three-generation adRP family with 13 affected patients. Six patients and five unaffected family members took part in the genetic study. The family exhibited incomplete penetrance, as the proband’s father was an asymptomatic mutation carrier. We calculated the LOD score in MLINK, and the result was 2.09. In most cases this would not be sufficient to prove linkage, but combined with prior evidence regarding *PRPF31* and this specific mutation, it is highly convincing. What is more, the change was absent in 200 unrelated, healthy Chinese controls, which together suggest that c.895T>C is a true mutation.

Patients carrying mutations in the *PRPF31* gene in this study demonstrated classic RP with night blindness as the initial symptom, followed by the gradual constriction of the visual field and a decline in visual acuity later in life. The fundi of the four probands were remarkably similar, with some variations in the amount of bone spicule pigmentation. The three deletions described in this study have a truncating effect on the protein and are likely to result in the degradation of the mRNA. Patients carrying a missense mutation, which would be expected to result in the mildest phenotype, are not necessarily affected more mildly. Our data also show that it is not always true that patients carrying a missense change will have the mildest phenotype. This strongly suggests that disease severity is not determined solely by *PRPF31* genotype; other genetic modifiers and/or environmental factors might influence phenotype expression. Therefore, the correlation between the genotype and the severity of the phenotype cannot be determined.

In conclusion, this study described three novel and one known mutation in the *PRPF31* gene in Chinese adRP patients. The identification of the genetic cause allows more precise genetic counseling, and in the future, this will be of importance when gene-specific or mutation-specific therapies become available.
